# Sex-Specific Isolation and Propagation of Human Premeiotic Fetal Germ Cells and Germ Cell-Like Cells

**DOI:** 10.3390/cells10051214

**Published:** 2021-05-16

**Authors:** Swati Mishra, Jasin Taelman, Yolanda W. Chang, Annekatrien Boel, Petra De Sutter, Björn Heindryckx, Susana M. Chuva De Sousa Lopes

**Affiliations:** 1 Ghent-Fertility and Stem Cell Team (G-FaST), Department for Reproductive Medicine, Ghent University Hospital, 9000 Ghent, Belgium; swati.mishra@gmail.com (S.M.); J.Taelman@lumc.nl (J.T.); Annekatrien.Boel@UGent.be (A.B.); Petra.DeSutter@UGent.be (P.D.S.); 2 Department of Anatomy and Embryology, Leiden University Medical Centre, 2333 ZC Leiden, The Netherlands; W.J.Chang@lumc.nl

**Keywords:** fetal germ cells, human embryonic stem cells, differentiation, sex-specific, FACS

## Abstract

The second trimester of human development is marked by asynchronous gonadal development hampering the isolation of homogenous populations of early and late fetal germ cells (FGCs). We evaluated the feasibility of using surface markers TNAP, PDPN, EPCAM and ITGA6 to isolate FGCs as well as human primordial germ cell-like cells (hPGCLCs) derived from embryonic stem cells (hESCs) from both sexes by fluorescence-activated cell sorting (FACS). Our results suggest that a combination of TNAP and PDPN was sufficient to separate populations of premeiotic FGCs and hPGCLCs in both sexes. This combination of antibodies also proved efficient in separating ‘mitotic’ from ‘retinoic-acid responsive’ female FGCs. Furthermore, we report that the differentiation efficiency of TNAP+PDPN+ hPGCLCs from hESCs was sex-independent, but the ability to propagate differed considerably between the sexes. In contrast to male, female hPGCLCs retained their characteristics and exhibited robust colony-forming ability when cultured for five days in medium containing LIF, forskolin and FGF2. We conclude that marked sex differences exist in the isolation and propagation of human FGCs and hPGCLCs. Our study provides novel insights relevant for the optimization of in vitro gametogenesis in humans.

## 1. Introduction

In vitro gametogenesis (IVG) is a promising avenue offering potential benefits both towards understanding germline development and factors contributing to infertility. While IVG may one day be used in human medically assisted reproduction, technical advancement is majorly dependent on understanding the markers and genes governing germline development. Primordial germ cells (PGCs) are known to be the earliest lineage-specific diploid progenitors of the germline in animals. The origin of human PGCs (hPGCs) is unclear, but they are likely specified around four to five weeks of gestation (WG) (equivalent to two to three weeks of development) in the posterior epiblast of post-implantation embryos around the onset of gastrulation [1,2]. Subsequently, hPGCs migrate and colonise the developing gonads around six to seven WG marking the end of the early phase of germ cell development [3]. Gonadal fetal germ cells (FGCs) respond to environmental stimuli to undergo proliferation and sex-determination, prompting sex-specific development. While human FGCs are known to be relatively homogenous until about 10 WG in females and males, progress to the second and third trimesters is pronouncedly asynchronous [4,5,6,7]. After 10 WG, male FGCs can be observed in at least two different stages (mitotic FGCs and mitotically-quiescent FGCs), and interestingly for a period of several weeks (10 to 16 WG) female hFGCs can also be observed in two main (premeiotic) stages (‘mitotic’ FGCs and ‘retinoic acid (RA)-responsive’ FGCs). In female, the innermost hFGCs will enter meiosis and by 20 WG female FGCs can be observed in at least six different stages (‘mitotic’ FGCs, ‘RA-responsive’ premeiotic FGCs and ‘meiotic’ cells in four stages of prophase I (leptotene, zygotene, pachytene, diplotene/dyctiate)) [4,5,6,7]. At birth, most female FGCs are in diplotene/dyctiate (oogonia) in primordial follicles and most male FGCs (prospermatogonia) are mitotically arrested [8,9].

Our understanding of markers to identify human FGCs at the various developmental stages during gestation has increased tremendously in recent years, due to the availability of single-cell transcriptomics data [7,10]. This has facilitated the search for suitable surface markers to isolate live populations of human (male and female) FGCs at specific stages of development [10,11,12], otherwise confounded due to their strong heterogeneity. One such example is the use of the surface marker KIT, which has been thoroughly used in studies to isolate hFGCs [7,12]. KIT is surely a useful surface marker, highly expressed by both premeiotic and diplotene female hFGCs [7,10], along with other somatic cells (such as endothelial cells) in the developing human gonad [3]. This inconveniences the use of KIT alone to separate different populations of hFGCs. However, a combination of TNAP (or ALPL) and KIT has been used to purify relatively homogenous population of hPGCs early during the first trimester (at seven weeks of development, equivalent to nine WG) [13].

Differentiation protocols generating human PGC-like cells (hPGCLCs) from human pluripotent stem cells (hPSCs), currently used to model early germline specification in vitro, are making it possible to obtain a sufficient number of hPGCLCs to perform a robust molecular characterization [14,15,16,17,18]. The generation of transgenic hPSCs, containing transgenes for known hPGC markers, such as PRDM1 (or BLIMP1), TFAP2C and NANOS3, has facilitated the optimization of differentiation protocols towards PGCLCs [14,15,16,17,18]. The use of transgenic hPSCs in combination with the use of certain surface makers shared by mouse and human PGCs, such as TNAP and KIT [12,13] has helped identify additional surface markers such as CD38, PDPN, EPCAM and ITGA6 suitable to purify hPGCLCs [11,14,16,19]. Interestingly, KIT is not routinely used to identify or purify hPGCLCs by FACS [11,14,20]. Other surface markers such as PDPN have been identified as suitable to identify hPGCs when combined with TNAP in immunohistochemical studies [21], but its presence on somatic cells prevents its use as a specific singular marker for early germ cells. PDPN is also expressed in hPGCLCs [14,16], but was only recently used to aid in the purification of hPGCLCs using FACS [19]. This solicits the need for identifying markers uniformly expressed in both premeiotic FGCs and hPGCLCs to be able to thoroughly compare populations and validate germline-differentiation protocols. Additionally, qualitative differences between female and male hPGCLCs have not been described, although male hESCs have shown higher efficiency than female hESCs to generate hPGCLCs [11].

A defining feature of hPGCLCs (and hPGCs) is their expression of pluripotency markers, such as TNAP, POU5F1 (or OCT4) and NANOG [14,16]. This has led to the hypothesis that it might be possible to propagate hPGCLCs under pluripotent culture conditions. Recently, a few studies have demonstrated the extended culture of hPGCLCs, that retained their identity for up to 21 days [22] as well as proliferative capacity for over four months [20]. Previous studies have tested culture conditions for the derivation of pluripotent human embryonic germ cells (hEGCs) from hPGCs in medium containing leukemia inhibitory factor (LIF), forskolin and fibroblast growth factor 2 (FGF2) (LFF medium) [23,24]. In addition, hPGCLCs are known to express markers of naïve pluripotency, such as *KLF4*, *TCL1B* and *TFCP2L1* [19], indicating the possibility of propagating hPGCLCs in naïve pluripotency medium. This led us to culture and evaluate the capacity of propagation of fluorescence-activated cell sorting (FACS)-sorted TNAP+PDPN+ (male and female) hPGCLCs short-term in LFF medium, in the germ cell specification competence-enhancing 4i medium (hESC medium supplemented with LIF, FGF2, TGFβ1 along with inhibitors for MEK, GSK3β, JNK and p38 [14] and the commercially available (naïve pluripotency stage) RSeT medium. In this study, we show comparable differentiation efficiency to TNAP+PDPN+ hPGCLCs between the female and male hESC lines used. However, only female hPGCLCs were able to further propagate in LFF medium, but not in 4i or RSeT medium, whereas male hPGCLCs were unable to propagate in all three conditions. Our study highlights a possible need to develop sex-specific protocols to propagate male and female hPGCLCs in culture.

## 2. Materials and Methods

### 2.1. Ethical Permissions

Differentiation of hPGCLCs from existing human embryonic stem cells (hESCs) was approved by the Institutional Review Board, Ghent University (EC2019/1595). Human fetal gonadal tissue was collected from elective abortions without medical indication, donated for research with signed informed consent of the patients. All procedures were approved by the Medical Ethical Committee of Leiden University Medical Centre (P08.087).

### 2.2. Culture of hESCs

Two different Activin A-derived primed hESC lines (U-11-4-A3; female and U-12-3-A3, male) [19] were maintained on a mitomycin-inactivated feeder layer of mouse embryonic fibroblasts (MEFs) in hESC medium (Gibco KnockOut-Dulbecco’s Modified Eagle Medium (KO-DMEM) (Thermo Fisher Scientific, Waltham, MA, USA), 20% Gibco KnockOut-serum replacement (KOSR) (Thermo Fisher Scientific), 1% Gibco Penicillin/Streptomycin (Thermo Fisher Scientific), 1% Gibco non-essential amino acids (NEAA) (Thermo Fisher Scientific), 0.4 mM Gibco L-Glutamine (Thermo Fisher Scientific), 0.1 mM Gibco β-mercaptoethanol (Thermo Fisher Scientific)) supplemented with 4 ng/mL FGF2 (Peprotech, Rocky Hill, NJ, USA) and 20 ng/mL Activin A (R&D Systems, Minneapolis, MN, USA). All cultures were maintained at 37 °C in hypoxic conditions (5% O_2_ and 6% CO_2_).

### 2.3. Differentiation of hESCs into hPGCLCs

The primed hESCs were differentiated to hPGCLCs as previously described [19]. Briefly, hESCs were transiently converted to the 4i-state by passing them six times in 4i medium (hESC medium supplemented with 20 ng/mL of recombinant human LIF (Peprotech), 8 ng/mL recombinant human FGF2, 1 ng/mL recombinant human TGFβ1 (Peprotech), 3 μM CHIR99021 (Axon Medchem, Groningen, The Netherlands), 1 μM PD0325901 (Cayman, Ann Arbor, MI, USA), 5 μM SB203580 (Biotechne, Minneapolis, MN, USA), 5 μM SP600125 (Tocris, Bristol, UK) and 10 µM ROCKi (Enzo Life Sciences, Farmingdale, NY, USA)) onto mitomycin-C-inactivated MEFs. To generate embryoid bodies (EBs), confluent wells of 4i-converted hESCs were dissociated using Gibco TrypLE Express (Thermo Fisher Scientific), pelleted at 750 rpm for 5 min, resuspended in 4i medium and plated onto 0.1% gelatine-coated plates for 1 h at 37 °C to allow removal of the (fast-attaching) MEFs. HESCs were resuspended at 65,000 cells/mL in hPGCLC differentiation medium (DM) consisting of Gibco Glasgow’s MEM (Thermo Fisher Scientific), 2 mM L-glutamine, 15% (*v*/*v*) KOSR, 1% P/S, 1% NEAA, 0.1 mM β-mercaptoethanol, 1 mM sodium pyruvate supplemented with 500 ng/mL Gibco recombinant human BMP4 (Thermo Fisher Scientific), 100 ng/mL Gibco recombinant human SCF (Thermo Fisher Scientific), 50 ng/mL recombinant human EGF (R&D Systems), 1μg/mL recombinant human LIF and 10µM of ROCKi. Next, 100μL/well cell suspension was added to 96-well U-bottomed low attachment plates (Corning, Corning, NY, USA) and centrifuged at 400× *g* for 2 min to facilitate the formation of EBs. The plates were incubated at 37 °C in hypoxic conditions for four days (D4) without medium changes. Differentiation experiments were performed three times (*N* = 3).

### 2.4. Human Fetal Gonads

Human fetal gonads isolated from different donors (female: 13 WG, 15 WG, 16 WG; male: 14 WG, 2 × 15 WG) were isolated in 0.9% NaCl (Fresenius Kabi, Bad Homburg, Germany) and dissociated with Accutase (Stemcell Technologies, Cambridge, UK) at 4 °C overnight (ON), followed by 30 min at 37 °C and cryopreserved in Bambanker (GC Lymphotech, Tokyo, Japan). The content of the cryovials was thawed, added to 10 mL DMEM with 15% Gibco fetal calf serum (FCS) (Thermo Fisher Scientific) and 10 µM ROCKi, and centrifuged at 300× *g* for 5 min. Pellets were resuspended in hESC medium, incubated for 30 min at 37 °C in hypoxia and used for FACS.

### 2.5. Fluorescence Activated Cell Sorting (FACS)

For FACS, D4 EBs were dissociated with TrypLE Express at 37 °C for 20 min with intermittent pipetting. Cell suspensions (human gonads or D4 EBs) were centrifuged at 300× *g* for 5 min and resuspended in 100 µL of FACS buffer (3% FCS in PBS with 10µM ROCKi) containing fluorescent-conjugated antibodies (Table S1) and incubated in the dark for 15 min at room temperature (RT). The cells were washed twice in FACS buffer, followed by resuspension in 100 µL FACS buffer with 1 μL 7AAD live/dead exclusion dye (BD Biosciences, San Francisco, CA, USA). This suspension was strained into 35 µm cell-strainer-snap-cap 5 mL FACS-tubes (Corning), followed by analysis and sorting on a FACSFusion cell sorter (BD Biosciences) using a 100 µm nozzle. The cells of interest were sorted directly into 1.5 mL tubes (Eppendorf, Hamburg, Germany) containing 1 mL hESC medium with 10 μM ROCKi. The gating strategy to purify live cells of interest is depicted in Figure S1a.

When intracellular markers were used (POU5F1), cells were first stained with membrane markers, then washed with PBS and fixed in 4% paraformaldehyde (PFA) (Sigma-Aldrich) for 10 min at RT. After washing with PBS, cells were permeabilized in 0.7% Triton-X (Sigma-Aldrich) for 15 min at RT. Finally, after washing with FACS buffer, cells were stained with the intracellular fluorescence conjugated antibody again at 4 °C by incubating for 30 min, washed with FACS buffer and analyzed on LSR-II flow cytometer (BD Biosciences). Flowcytometry data were collected with FACSDiva Software (BD Biosciences) and analyzed with FlowJo v10.7.1 (BD Biosciences).

### 2.6. Extended Culture of hPGCLCs

Mitomycin-inactivated MEFs were seeded on round glass coverslips in 12-well plates and cultured ON in three types of media: 4i medium, RSET medium (Stemcell Technologies) or LFF medium (hESC medium supplemented with 4 ng/mL FGF2, 10 µM forskolin (Sigma-Aldrich, St. Louis, MO, USA) and 1000 U/mL human LIF) [24] containing 10 µM ROCKi. The FACS-sorted (PDPN+TNAP+) hPGCLCs from D4 EBs were centrifuged at 1600 rpm for 5 min and resuspended in fresh hESC medium with 10µM ROCKi. Next, 1000 cells/well were added to the MEF-containing wells with 4i, RSET or LFF media and cultured in hypoxic conditions at 37 °C in for two or five days, with medium refreshment every other day. Extended culture experiments were performed three times (*N* = 3).

### 2.7. Immunofluorescence, Imaging and Quantification

The EBs and coverslips with hESCs or hPGCLC extended cultures were fixed with 4% PFA (Sigma-Aldrich) for 1 h at RT. Fixed samples were washed in 0.1% bovine serum albumin (BSA) (Merck Millipore, Burlington, MA, USA), in PBS and permeabilized with 0.1% Triton-X100 (Sigma-Aldrich) in PBS for 8 min (hESCs/hPGCLCs) or 0.5% Triton-X100 in PBS for 1 h (EBs). Samples were then blocked for 2 h in 10% FCS and 0.5% BSA in PBS followed by incubation with primary antibodies (Table S1) ON at 4 °C and secondary antibodies (Table S1) for 2 h at RT. After washing, samples were counterstained with 4′,6-diamidino-2-phenylindole (DAPI) (Thermo Fisher Scientific) for 10 min and mounted on glass slides in mounting medium containing 2.4% DABCO (Sigma-Aldrich) in glycerol (Novolab, Geraardsbergen, Belgium). Samples were imaged on a SP8 confocal microscope (Leica, Wetzlar, Germany) using SPX software. The obtained images were analyzed using FIJI (ImageJ) version 2.1.0 [25].

Fetal gonads were embedded in paraffin using a Shandon Excelsior tissue processor (Thermo Fisher Scientific) and sectioned (5 μm) using a RM2065 microtome (Leica) onto StarFrost slides (Waldemar Knittel, Brunswick, Germany). To deparaffinize the sections, paraffin sections were treated with xylene and rehydrated in a dilution series of ethanol and water for the last step. After rehydration, antigens were retrieved by incubating sections with 0.01 M citric buffer (pH 6.0) for 12 min at 98 °C in a TissueWave 2 microwave (Thermo Fisher Scientific) and allowed to cool down. After rinsing with PBS, sections were incubated for 1 h at RT with blocking solution (1% BSA and 0.05% Tween-20 (Merck)) and treated with primary antibodies ON at 4 °C. Sections were washed three times with PBS with 0.05% Tween-20 (PBST), incubated with secondary antibodies (Table S1) and DAPI for 1 h at RT, washed three times with PBS and mounted with coverslips using ProLong Gold (Thermo Fisher Scientific). Slides were imaged with a LSM 900 Airyscan 2 confocal laser scanning microscope (Zeiss, Jena Germany), and greyscale single channel images were combined and edited (adjustment of brightness/contrast) in Photoshop v21.2.4 (Adobe, San Jose, CA, USA).

For quantification of FGCs, male and female gonadal sections were scanned on a Panoramic MIDI digital scanner (3DHISTECH Ltd., Budapest, Hungary); image analysis was carried out using IMAGEJ v2.1.0/1.53c. Cell counts were made in three to four large areas in the cortex of several gonadal sections using the multipoint tool and the cell counter plugin. The percentage of different populations of FGCs were calculated on the total number of FGCs. Graphs depicting the individual percentage per area (circles) as well as the mean ± standard deviation were plotted using GraphPad Prism version 8.4.1.

### 2.8. Analysis of RNA Sequencing (RNASeq) Data

Single cell RNASeq data from human fetal gonads ((unique molecular identifier (UMI) count data (GSE86146) and cell type metadata (Final_clusters)) [7] was analysed in R (v4.0.2). Cells with <2000 genes or >100,000 total counts were excluded from further analysis. Cells from 14 weeks were also excluded from the analysis. Transcripts per million (TPM) values were calculated as counts per gene/(total counts per cell) × 10^6^. TPM values were normalized by log2(TPM + 1) transformation. The data was filtered to include only male and female FGCs and somatic cell (soma) clusters. For those clusters, the mean expression of each gene of interest was calculated using the R function rowMeans. The mean gene expression per cluster was visualized by heatmap using the Pheatmap-package (v1.0.12), using euclidian distance-based clustering.

For the comparison of male and female ‘mitotic FGC’ cell clusters, we first generated a Seurat (v3.2.2) object from all unnormalized TPM values, including only genes with an expression level > 1 in at least 10 cells. NormalizeData was used to log-normalize the data, with a scale factor of 10^6^. Using FindVariableFeatures, the 2000 most variable genes were calculated, with vst as the selection method. The ‘mitotic’ male and female FGCs were as originally defined by the authors [7]. Next, differentially expressed genes (DEGs) were calculated specifically for male versus female ‘mitotic’ FGCs, using the Seurat function FindMarkers, only retaining genes detected in at least 60% of either cell cluster. Genes with an ln (average fold change) > 0.693 (or a fold change > 2) and a Bonferroni-adjusted *p*-value < 0.05 were considered to be significantly differential (Table S2). A volcano plot was generated to visualize significant DEGs using ggplot2 (v3.3.2).

## 3. Results

### 3.1. Surface Markers to Separate POU5F1^Hi^DDX4^Lo^ from POU5F1^Lo^DDX4^Hi^ Premeiotic hFGCs

**Figure 1:**
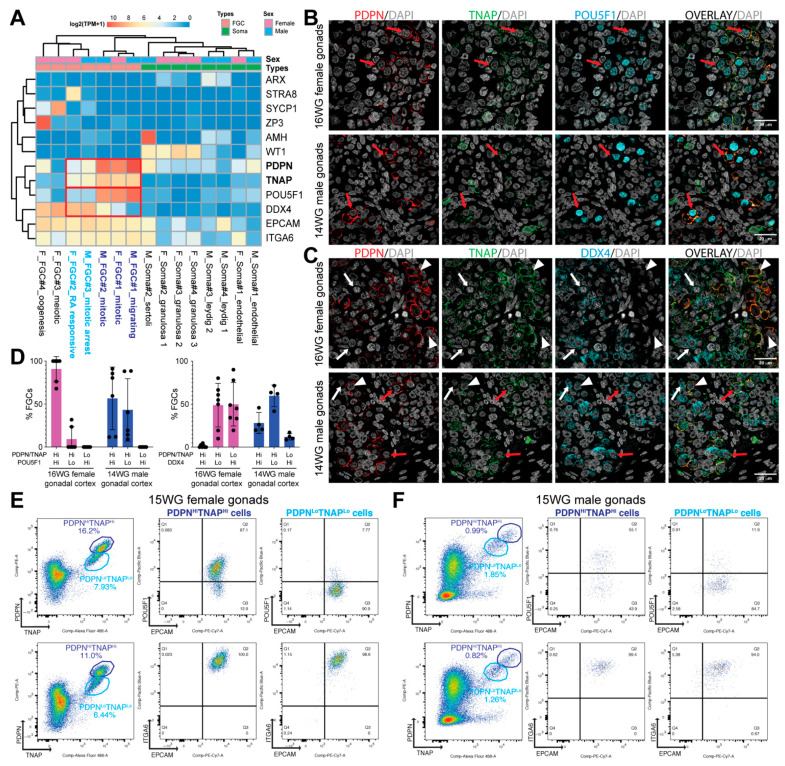
Expression of cell surface markers in male and female human fetal gonads. (**A**) Heatmap generated using existing data [7] showing expression of several genes of interest in male and female fetal germ cells (FGCs) and gonadal somatic cells (soma). (**B**) Histological sections of 16 WG female gonads and 14 WG male gonads immunostained for PDPN, TNAP and POU5F1. Red arrows indicate cells positive for PDPN, TNAP and POU5F1. Scale bars are 20 μm. (**C**) Histological sections of 16 WG female gonads and 14 WG male gonads immunostained for PDPN, TNAP and DDX4. Red arrows indicate cells positive for PDPN, TNAP and DDX4, white arrows indicate cells positive for DDX4; arrowheads indicate cells positive for PDPN and TNAP. Scale bars are 20 μm. (**D**) Quantification of male and female FGCs in 16 WG female gonads and 14 WG male gonads regarding expression of PDPN, TNAP and POU5F1 (left) and PDPN, TNAP and DDX4 (right). The individual percentages are given (black circles) as well as mean±standard deviation. (**E**) FACS plots showing expression of PDPN/TNAP in female 15 WG gonads. The expression of POU5F1/EPCAM (top panel) and ITGA6/EPCAM (bottom panel) are shown for the PDPN/TNAP high (Hi) and low (Lo) populations. (**F**) FACS plots showing expression of PDPN/TNAP in male 15 WG gonads. The expression of POU5F1/EPCAM (top panel) and ITGA6/EPCAM (bottom panel) are shown for the PDPN/TNAP high (Hi) and low (Lo) populations.

We analysed the gene expression levels of surface markers *EPCAM*, *ITGA6*, *PDPN* and *TNAP* on human FGCs and somatic niche (soma) at various stages of development using an online available single-cell transcriptomics data (RNASeq) [7]. High expression of *TNAP* and *PDPN* coincided with high levels of *POU5F1* in male and female ‘mitotic’ and ‘migrating’ FGC clusters (Figure 1A). In addition, intermediate/low expression of *TNAP* and *PDPN* coincided with high levels of *DDX4* (or *VASA*) and low levels of *POU5F1* in male and female late FGC clusters (‘mitotic arrest’ male FGCs and *STRA8*+ ‘RA-responsive’ female FGCs), and *TNAP* was not observed in female meiotic FGCs clusters (*SYCP1*+ ‘meiotic’ and *ZP3*+ ‘oogenesis’) (Figure 1A). We validated the expression of TNAP and PDPN in human 16 WG female and 14 WG male gonads (Figure 1B–D). This gestational age was chosen to avoid the presence of meiotic cells in female gonads [5], that would confound the analysis of premeiotic FGCs. As expected, female FGCs strongly positive for DDX4 were low in PDPN/TNAP, and female FGCs strongly positive for PDPN/TNAP were also POU5F1^Hi^ (Figure 1B–D). In contrast to females where two FGC populations were observed, in males, three populations could be distinguished: FGCs strongly positive for PDPN/TNAP were either POU5F1^Hi^ or DDX4^Hi^ and FGCs lowly positive for PDPN/TNAP were DDX4^Hi^ (Figure 1B–D).

*EPCAM* and *ITGA6* were expressed at comparable levels in all FGCs in both sexes (Figure 1A), suggesting that a combination of EPCAM and ITGA6 that mark all FGCs is not suitable to separate different types of FGCs in either males or females.

### 3.2. Isolation of POU5F1^Hi^ and POU5F1^Lo^ Premeiotic hFGCs Using FACS

We examined the capacity of PDPN and TNAP to FACS isolate premeiotic hFGCs in 15 WG gonads of both sexes (Figure 1E,F; Figure S1b). In agreement with the transcriptional data and immunofluorescence, the FACS analysis revealed two distinct populations of PDPN- and TNAP-positive cells: showing high levels (PDPN^Hi^TNAP^Hi^) and low levels (PDPN^Lo^TNAP^Lo^) of expression (Figure 1E,F).

In 15 WG female gonads, the PDPN^Hi^TNAP^Hi^ population was highly enriched in POUF51+ cells (87.1%), whereas the PDPN^Lo^TNAP^Lo^ only contained 7.7% cells of POUF51+ cells, probably due to the proximity of the two clusters (Figure 1E). Both clusters showed a high expression of EPCAM and ITGA6 (Figure 1E). Our data suggested that different levels of PDPN and TNAP may be sufficient to isolate the two populations of premeiotic female hFGCs: ‘mitotic’ (POU5F1^Hi^DDX4^Lo^) FGCs and ‘*STRA8*+ RA-responsive’ (POU5F1^Lo^DDX4^Hi^) FGCs.

The 15 WG male gonads also showed PDPN^Hi^TNAP^Hi^ cells and PDPN^Lo^TNAP^Lo^ cells (Figure 1F); however, the cluster separation was less pronounced than in females of similar gestational age. In agreement with the results obtained from immunofluorescence (Figure 1C,D), PDPN^Hi^TNAP^Hi^ cells showed a pronounced heterogeneity regarding the expression of POU5F1 (Figure 1F). Both PDPN^Hi^TNAP^Hi^ and PDPN^Lo^TNAP^Lo^ cells were highly positive EPCAM and ITGA6 (Figure 1F) suggesting they were bonafide FGCs.

In conclusion, a combination of PDPN and TNAP immunostaining is effective to separate premeiotic hFGCs in both sexes, but different levels of PDPN and TNAP are able to separate ‘mitotic’ (POU5F1^Hi^DDX4^Lo^) from ‘*STRA8*+ RA-responsive’ (POU5F1^Lo^DDX4^Hi^) FGCs in female gonads. However, for male hFGCs, additional surface marker combinations should be investigated to separate FACS ‘mitotic’ (POU5F1^Hi^DDX4^Lo^) from ‘mitotic-arrest’ (POU5F1^Lo^DDX4^Hi^) hFGCs.

### 3.3. TNAP and PDPN Are Suitable to Isolate Male and Female hPGCLCs by FACS

**Figure 2:**
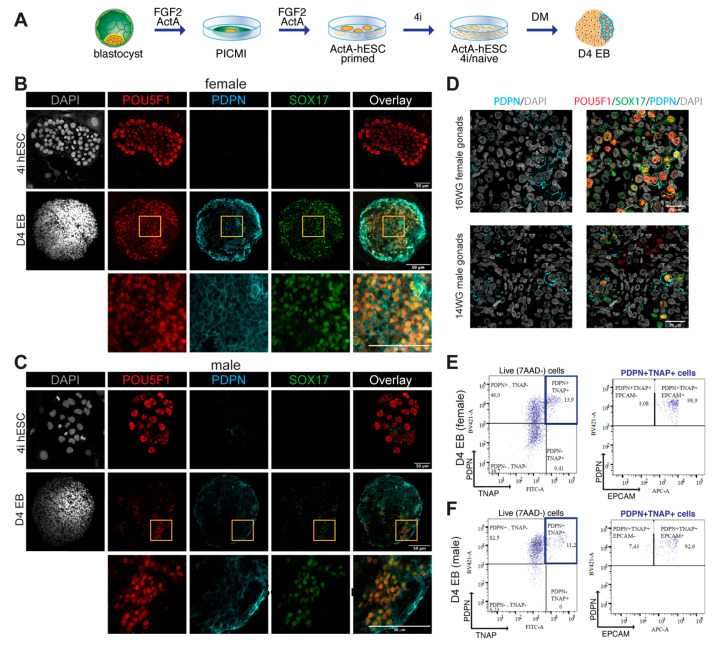
Differentiation of hPGCLCs from Activin A-derived hESCs. (**A**) Scheme showing the differentiation of hPGCLCs from Activin A-derived hESCs. Human blastocyst embryos were plated in medium containing FGF2 and Activin A (ActA), the resulting post-inner cell mass intermediate (PICMI) was further expanded in the same medium to derive primed ActA-hESCs. After several passages in 4i medium, the ActA-hESCs were differentiated to hPGCLCs using differentiation medium (DM) using an embryoid body (EB)-assay for four days. (**B**) Female ActA-derived hESC (U-11-4-A3) adapted to 4i-condition (top panels) and D4 EBs (middle panels) immunostained for POU5F1, SOX17 and PDPN. Orange boxes indicate regions of the EBs magnified (bottom panels). Scale bars are 50 µm. (**C**) Male ActA-derived hESC (U-12-3-A3) adapted to 4i-condition (top panels) and D4 EBs (middle panels) immunostained for POU5F1, SOX17 and PDPN. Orange boxes indicate regions of the EBs magnified (bottom panels). Scale bars are 50 µm. (**D**) Histological sections of 16 WG female gonads and 14 WG male gonads immunostained for POU5F1, PDPN and SOX17. Scale bars are 20 μm. (**E**) FACS plots showing the expression of TNAP, PDPN and EPCAM in live cells from female D4 EBs (U-11-4-A3). (**F**) FACS plots showing the expression of TNAP, PDPN and EPCAM in live cells from male D4 EBs (U-12-3-A3).

Human PGCLCs can be obtained from primed hESCs by first converting them to the 4i state of pluripotency followed by differentiation as EBs for four days in hPGCLC-differentiation medium containing BMP4, hLIF, SCF, EGF and ROCKi (DM) [14]. We have recently shown that the efficiency can be further enhanced three- to fourfold using hESCs that have been initially derived in the presence of Activin A [19] and we differentiated Activin A-derived female (U-11-4-A3) and male (U-12-3-A3) lines to hPGCLCs (Figure 2A). HPGCLCs co-express POU5F1 and SOX17, whereas POU5F1+ hESCs are negative for SOX17 [14,19]. In agreement, both female (Figure 2B) and male 4i-hESCs (Figure 2C) showed high expression of POU5F1 and no expression of SOX17, whereas hPGCLCs in D4 EBs showed strong colocalised expression of POU5F1 and SOX17 (Figure 2B,C). PDPN was lowly expressed in 4i-hESCs and highly expressed in D4 EBs in male and female POUF51+SOX17+ hPGCLCs (Figure 2B,C). As a positive control, 15 WG female and male gonads also showed strong colocalization of POU5F1, SOX17 and PDPN in ‘mitotic’ (POU5F1^Hi^DDX4^Lo^) FGCs in both sexes (Figure 2D) and the negative controls for immunofluorescence are provided in Figure S1c,d.

Next, we investigated by FACS the percentage of PDPN+TNAP+ hPGCLCs present in D4 EBs derived from female and male PSCs. The use of PDPN in FACS, together with TNAP, is important to ensure that any remaining hESCs that are PDPN—(Figure 2B,C) but TNAP+, present in D4 EBs are excluded. We observed a comparable percentage (13.9% in female; 11.2% in male) (Figure 2E,F; Figure S1e). As expected, the great majority of (male and female) PDPN+TNAP+ hPGCLCs were also EPCAM+ (98.9% in female; 92.6% in male) (Figure 2E,F).

### 3.4. Male and Female hPGCLCs Propagate Differently in LFF Medium

**Figure 3:**
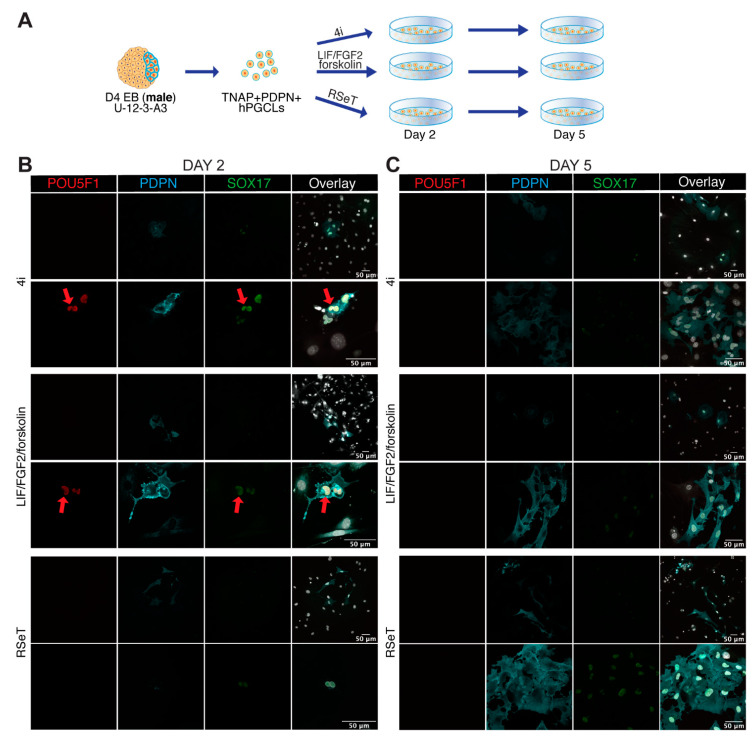
Evaluating the propagation of male hPGCLCs in vitro. (**A**) Scheme showing the extended culture protocol used of (male) TNAP+PDPN+ hPGCLCs FACS-isolated from D4 EBs and cultured in different media (4i, RSeT and LIF/FGF2/forskolin). (**B**) Images from day 2 of extended culture of male hPGCLCs in different media (4i, RSeT and LIF/FGF2/forskolin) immunostained for POU5F1, SOX17 and PDPN. Red arrows point to POU5F1+SOX17+PDPN+ hPGCLCs. Scale bars are 50 µm. (**C**) Images from day 5 of extended culture of male hPGCLCs in different media (4i, RSeT and LIF/FGF2/forskolin) immunostained for POU5F1, SOX17 and PDPN. Scale bars are 50 µm.

**Figure 4:**
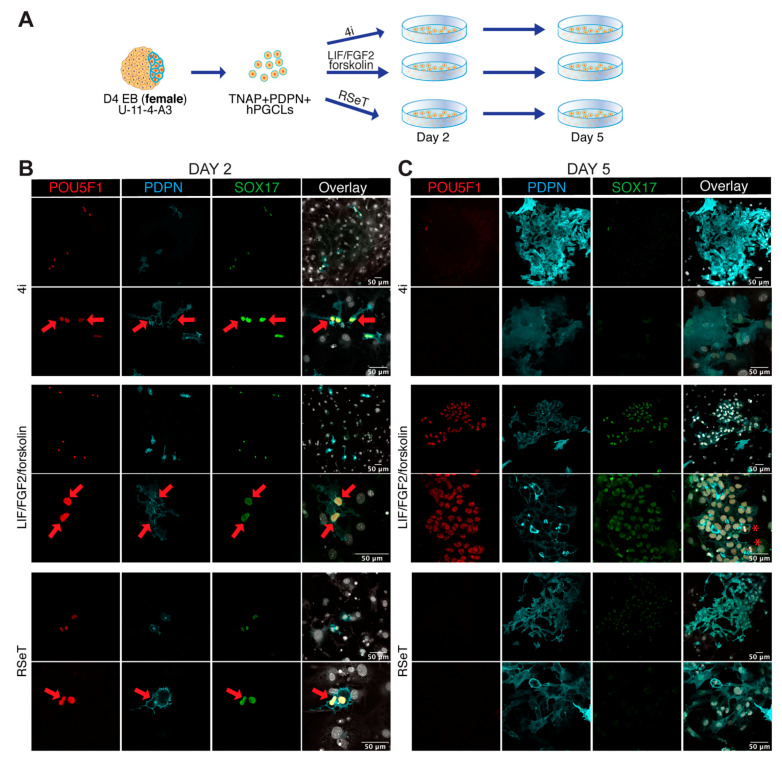
Evaluating the propagation of female hPGCLCs in vitro. (**A**) Scheme showing the extended culture protocol used of (female) TNAP+PDPN+ hPGCLCs FACS-isolated from D4 EBs and cultured in different media (4i, RSeT and LIF/FGF2/forskolin). (**B**) Images from day 2 of extended culture of female hPGCLCs in different media (4i, RSeT and LIF/FGF2/forskolin) immunostained for POU5F1, SOX17 and PDPN. Red arrows point to POU5F1+SOX17+PDPN+ hPGCLCs. Scale bars are 50 µm. (**C**) Images from day 5 of extended culture of female hPGCLCs in different media (4i, RSeT and LIF/FGF2/forskolin) immunostained for POU5F1, SOX17 and PDPN. Red asterisks mark dividing cells. Scale bars are 50 µm.

To further investigate differences between male and female hPGCLCs differentiated from Activin A-derived female (U-11-4-A3) and male (U-12-3-A3) lines, we cultured FACS-sorted TNAP+PDPN+ hPGCLCs from male and female D4 EBs in different media (4i, RSeT and LFF) for two and five days (Figure 3 and Figure 4). The time points were selected to monitor attachment and survival (day 2) and colony formation (day 5).

After male TNAP+PDPN+ hPGCLCs were cultured for two days, we observed cells that showed colocalization of nuclear POU5F1 and SOX17 with PDPN in the cell surface, most likely corresponding to the FACS-sorted hPGCLCs, in 4i and LFF medium, but not in RSeT medium (Figure 3B). Interestingly, those cells showed the characteristic kidney-shaped nuclear morphology of germ cells cultured on tissue-culture plastic [2,26,27]. However, by day 5 in culture, the cells had lost POU5F1 expression, stained weakly for SOX17 and PDPN had leaked into the cytoplasm, although some degree of proliferation was observed in all three media (4i, RSeT and LFF) (Figure 3C). This indicated that the male cells survived in the three media for five days, but were losing their hPGCLCs characteristics. Cytoplasmic PDPN has been previously reported in certain cancer cell types [28] and could reflect cellular malfunction regarding the segregation of the protein to the cell surface.

In contrast to male, many female TNAP+PDPN+ hPGCLCs showed strong colocalization of nuclear POU5F1 and SOX17 with PDPN in the cell surface in all 4i, LFF and RSeT media at day 2 (Figure 4B). As with the male cells, the female cells also showed the characteristic nuclear kidney shape and, by day 5, female cells were also losing their hPGCLCs characteristics when cultured in 4i and RSeT media (Figure 4C). However, when cultured in LFF medium, female hPGCLCs formed thriving colonies of proliferating POU5F1+SOX17+PDPN+ cells.

Together, we suggest that male and female hPGCLCs may require different culture conditions to be propagated in vitro. Although it is likely that hPGCLCs represent a developmental stage prior to sex-determination, hPGCLCs could already have transcriptional differences, possibly linked to the sex chromosomes, that could explain the different behaviour in culture. In this regard, we have compared female and male ‘mitotic’ (POU5F1^Hi^DDX4^Lo^) hFGCs that are to a certain extent the in vivo counterparts of hPGCLCs. We provide a volcano plot showing the most significant gene expression differences, mostly Y-linked (in male hFGCs) (Figure S1f), and a list of differentially expressed genes (Table S2), suggesting that ‘mitotic’ (POU5F1^Hi^DDX4^Lo^) hFGCs show many sex-specific transcriptional differences already at this developmental stage. Further analysis of the transcriptional profile of male and female hPGCLCs remains to be investigated.

## 4. Discussion

The systematic study of human germline development through analysis of in vivo tissue and in vitro models could aid in understanding the causes and possible treatments for human infertility. Studying the cellular and molecular mechanisms that govern human germline specification and development has been challenging due to the impracticality of accessing early human post-implantation embryos and the limited availability of human embryonic and fetal tissue [29]. Therefore, germline studies are being routinely modelled in vitro using hPSCs directed to differentiate into hPGCLCs [30,31]. Apart from providing valuable data to understand determinants of germ cell specification including identification of previously unknown early germ cell markers, this model has been instrumental in driving comparative studies of transcriptional networks that govern germline specification in human, mice, cynomolgus monkey and porcine models [32,33,34,35]. However, the application of gene expression data derived from the relatively synchronous hPGCLCs reflective of ‘mitotic’ FGCs [14] is still rather limited compared to the asynchronously developing FGCs during the second trimester [3]. Hence, identifying suitable cell surface markers to reflect the various stages of development of FGCs will warrant the discovery of a different set of markers than those used to purify hPGCLCs and ‘mitotic’ FGCs. Our data suggests that the combination of PDPN and TNAP is not only adequate to purify hPGCLCs in the context of EBs [19], but we also demonstrate that the two main types of (premeiotic) female FGCs (‘mitotic’ and ‘RA-responsive’ FGCs) expressed different levels of PDPN and TNAP and could be efficiently separated or purified using this combination of antibodies in FACS. This is the first time that a set of two surface markers has been shown to efficiently separate these two states of (premeiotic) female hFGCs. In contrast to females, the two stages of premeiotic male hFGCs could not be efficiently separated using a combination of PDPN and TNAP, highlighting a sex-specific difference and the need to further investigate sex-specific differences that could confound the analysis of early in vitro gametogenesis events in humans.

In this study, we show that female (TNAP+/PDPN+) hPGCLCs differentiated from Activin A-derived hESCs could be propagated for up to five days in LFF medium and retained co-expression of POU5F1, SOX17 and PDPN. LFF media has been previously used in attempts to derive embryonic germ cells from both male and female FGCs isolated from human gonads of the first trimester [24]. However, we were unable to obtain similar results for hPGCLCs derived from male hESCs. These outcomes suggest sex-specific differences in the requirements for extended propagation of hPGCLCs, although further validation and characterization is necessary. Alternatively, the results observed could be reflective of the Activin A-derivation protocol, clonality or the genetic background of the two hESC lines used to induce hPGCLCs. Hence, the use of additional Activin A-derived hESC lines will be necessary to thoroughly consolidate and clarify the observed sex-specific differences regarding propagation in LFF medium. Moreover, while we show colony formation and survival of cells at day 5, further characterisation and extended culture are needed to examine the identity of both female and male cells.

The efficient differentiation of a large number of hPGCLCs from hPSCs is currently possible using several published protocols. One example is a protocol using hESCs derived in Activin A to increase hPGCLC yield [19], while another example is expansion through recurrent FACS-sorting of hPGCLCs in long-term culture [20]. In contrast to our study, Murase et al. succeeded in expanding male hPGCLCs. Comparing the medium used by Murase with the LFF media reveals similar supplementation with 10 μM Forksolin and 1000U/mL of hLIF; however, Murase uses 20 ng/mL FGF2 (opposed to 4ng/mL in LFF) as well as supplementation with 50 ng/mL EGF and 100 ng/mL of SCF. In addition, Murase adds 2.5% FCS to provide additional support to the growing cells, whereas LFF only contains KOSR. It remains to be investigated whether the medium used by Murase also supports the expansion of female hPGCLCs.

While currently, most widely used protocols to differentiate hPSCs into hPGCLCs are carried out independent of MEFs [14,16], both our study and the study by Murase [20] expand the hPGCLCs on MEFs. MEFs are known to produce factors such as FGF2 that regulate, among others, the expression of key members of the TGFβ pathway and promote self-renewal in hPSCs [36]. Hence, factors secreted by the MEFs, and in particular FGF2, may have an important influence on the (long-term) maintenance of hPGCLCs.

Optimized protocols that contribute to efficiently generating a high number of hPGCLCs will allow a robust genome-wide epigenetic analysis (which often requires a large amount of material as input, such as bisulphite sequencing or chromatin immunoprecipitation), contributing to elucidate events that occur during early FGC development. In the future, suitable extended hPGCLC culture conditions, perhaps with the possibility to co-culture with the human fetal gonadal cells, may allow us to mimic and understand further developmental steps in human gametogenesis, such as sex-specific morphological development, meiotic entry and eventual maturation into mature gametes.

## 5. Conclusions

This study addresses the challenges of the sex-specific characterization of early hFGCs and hPGCLCs and identifies the markers TNAP and PDPN as a suitable combination to purify this population from premeiotic hFGCs in both sexes. Moreover, we show that TNAP and PDPN is also effective in separating ‘mitotic’ from ‘RA-responsive’ female FGCs. In contrast, this is not as effective in separating ‘mitotic’ from ‘mitotically-arrested’ male FGCs, suggesting a pronounced sex-specific difference. This study also shows that female hPGCLCs differentiated from Activin A-derived hESCs could be propagated for up to five days while retaining their identity, in contrast to male hPGCLCs. This suggests that hPGCLCs (and their in vivo counterparts early FGCs) may already exhibit sex-specific features, that may be determinant for successful extended propagation and further in vitro gametogenesis.

## Data Availability

This study did not generate novel datasets. The accession numbers for the datasets used in this study are NCBI Gene Expression Omnibus (GEO): GSE86146.

## Supplementary Materials

The following are available online at https://www.mdpi.com/article/10.3390/cells10051214/s1. Figure S1: Gating strategies, negative controls and volcano plot. (A) FACS plots showing the gating strategy used for the analysis of the different cell populations in D4 EBs. (B) FACS plots showing the gating of the unstained controls on 15 WG female gonads (left panels) and 15 WG male gonads (right panels). (C) Negative control for whole mounts (DAPI and secondary antibodies only) for 4i-hESCs (top panel) and D4 EBs (bottom panel). Scale bars are 50 μm. (D) Negative control for paraffin sections (DAPI and secondary antibodies only) from 14 WG male gonads. Scale bars are 20 μm. (E) FACS plots showing the gating of the unstained controls on live cells from D4-EBs. (F) Volcano plot showing differentially expressed genes between male (M) mitotic FGCs (#2) and female (F) mitotic FGCs (#1). Table S1: list of antibodies used for immunofluorescence and FACS. Table S2: list of differentially expressed genes between male mitotic and female mitotic FGCs.
