# Supplementary material for: Sex-Specific Isolation and Propagation of Human Premeiotic Fetal Germ Cells and Germ Cell-Like Cells
Source: Cells. 2021 May 16;10(5):1214. doi: 10.3390/cells10051214 (PMC8156680; doi:10.3390/cells10051214)
Supplement: Supplementary file 1 [file cells-10-01214-s001.zip › cells-1112689-supplementary.pdf]

Supplementary Information

Sex-Specific Isolation and Propagation of Human Premeiotic Fetal Germ Cells and Germ Cell-Like Cells

Swati Mishra [1], Jasin Taelman [1], Yolanda W. Chang [2], Annekatrien Boel [1], Petra De Sutter [1], Björn Heindryckx [1,*,†] and Susana M. Chuva De Sousa Lopes [1,2,*,†]

[1] Ghent-Fertility and Stem Cell Team (G-FaST), Department for Reproductive Medicine, Ghent University Hospital, 9000 Ghent, Belgium; swati.mishra@gmail.com (S.M.); J.Taelman@lumc.nl (J.T.); Annekatrien.Boel@UGent.be (A.B.); Petra.DeSutter@UGent.be (P.D.S.)

[2] Department of Anatomy and Embryology, Leiden University Medical Centre, 2333 ZC Leiden, The Netherlands; W.J.Chang@lumc.nl

* Correspondence: Bjorn.Heindryckx@ugent.be (B.H.); lopes@lumc.nl (S.M.C.D.S.L.); Tel.: +32-9332-4748 (B.H.); +31-71-526-9350 (S.M.C.D.S.L.)

† These two authors contributed equally to this manuscript.

List of Supplementary items:

- Figure S1
- Table S1
- Table S2

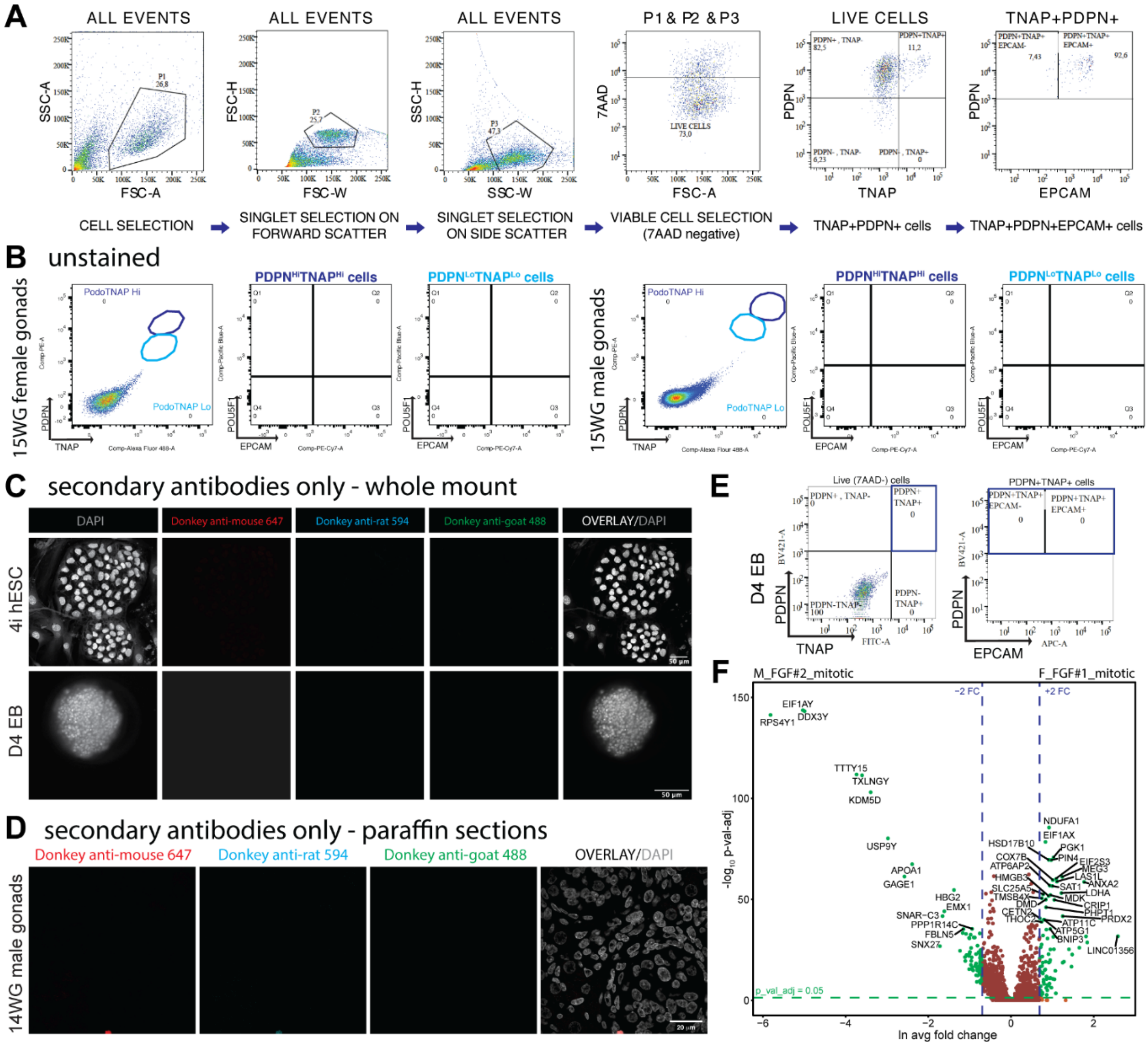

Figure S1: Gating strategies, negative controls and volcano plot. A) FACS plots showing the gating strategy used for the analysis of the different cell populations in D4 EBs. **B)** FACS plots showing the gating of the unstained controls on 15WG female gonads (left panels) and 15WG male gonads (right panels). **C)** Negative control for whole mounts (DAPI and secondary antibodies only) for 4i-hESCs (top panel) and D4 EBs (bottom panel). Scale bars are 50µm. **D)** Negative Control for paraffin sections (DAPI and secondary antibodies only) from 14WG male gonads. Scale bars is 20µm. **E)** FACS plots showing the gating of the unstained controls on live cells from D4-EBs. **F)** Volcano plot showing differentially expressed genes between male (M) mitotic FGCs (#2) and female (F) mitotic FGCs (#1).

Table S1. List of antibodies used for immunofluorescence and FACS.

Antibody	Host Species	Stock Concentration	Dilution used	Company / Product No.
**Primary antibodies for immunofluorescence**				
POU5F1 (OCT3/4)	mouse anti-human	0.2mg/ml	1:100	Santa Cruz / SC-5279
SOX17	goat anti-human	0.2mg/ml	WM: 1:175<br>IHC: 1:200	R&D Systems / AF1924
PDPN	rat anti-human	0.5mg/ml	WM: 1:200<br>IHC: 1:500	eBiosciences / 14-9381-80
TNAP (ALPL)	goat anti-mouse	0.2mg/ml	1:200	R&D system / AF2910
DDX4	rabbit anti-human	1mg/ml	WM: 1:200<br>IHC: 1:500	Abcam / Ab13840
**Secondary antibodies for immunofluorescence**				
Alexa 488	donkey anti-goat	2mg/ml	1:500	Abcam / ab150129
Alexa 594	donkey anti-rat	2mg/ml	1:500	Invitrogen / A21209
Alexa 647	donkey anti-mouse	2mg/ml	1:500	Invitrogen / A31571
Alexa 647	donkey anti-rabbit	2mg/ml	1:500	Invitrogen / A31573
**Conjugated antibodies for FACS**				
BV421 conj. PDPN	mouse anti-human	0.2mg/ml	1:100	BD Biosciences / 566456
PE conj. PDPN	rat anti-human	0.05mg/ml	1:3000	eBioscience / 12-9381-80
Alexa 488 conj. TNAP	mouse anti-human	1ml in 100ml of staining volume (for 250K cells)	1:100	BD Biosciences / 561495
BV421 conj. ITGA6	mouse anti-human	1ml in 100ml of staining volume (for 250K cells)	1:100	BioLegend / 313624
APC conj. EPCAM	mouse anti-human	1ml in 100ml of staining volume (for 250K cells)	1:100	BioLegend / 324207
PE/Cyanine7 conj. EPCAM	mouse anti-human	1ml in 100ml of staining volume (for 250K cells)	1:100	BioLegend / 324221
BV421 conc. POU5F1	mouse anti-human	0.2mg/ml	1:100	BD Biosciences / 565644

Table S2. List of differentially expressed genes between male mitotic and female mitotic FGCs.

	p_val	ln avg fold change	pct.1	pct.2	p_val_adj
RPS4Y1	2.245E−146	−5.815331595	0.033	0.997	4.524E−142
EIF1AY	9.779E−149	−5.031866128	0.007	0.997	1.97E−144
DDX3Y	2.888E−148	−4.992315905	0.005	0.994	5.819E−144
TTTY15	6.79E−117	−3.73437114	0.005	0.843	1.368E−112
TXLNGY	1.716E−116	−3.601192326	0.007	0.843	3.458E−112
KDM5D	4.374E−108	−3.3932949	0.002	0.795	8.812E−104
USP9Y	3.0382E−85	−2.975198277	0	0.663	6.1211E−81
GAGE1	2.486E−66	−2.577180458	0.042	0.639	5.0084E−62
APOA1	1.962E−72	−2.390622397	0.044	0.663	3.9528E−68
SNAR−C4	7.7607E−32	−1.718893356	0.314	0.678	1.5635E−27
SNAR−C3	1.049E−46	−1.655379224	0.759	0.979	2.1134E−42
EMX1	4.4218E−49	−1.612674343	0.096	0.617	8.9085E−45
HBG2	1.2471E−59	−1.377466958	0.094	0.645	2.5125E−55
PRTG	1.5742E−29	−1.232335501	0.208	0.602	3.1715E−25
ZADH2	2.4357E−38	−1.167510598	0.183	0.672	4.9071E−34
FBLN5	2.6929E−40	−1.149191149	0.494	0.934	5.4254E−36
S100A11	2.1599E−26	−1.111847619	0.344	0.744	4.3516E−22
LOC100996255	1.7205E−24	−1.103450201	0.478	0.78	3.4664E−20
KRCC1	6.5745E−38	−1.086252179	0.19	0.684	1.3246E−33
SLC35D2	1.9228E−27	−1.074560572	0.398	0.786	3.874E−23
SPRY4	5.1802E−27	−1.027175727	0.419	0.792	1.0437E−22
ENPP4	6.4306E−36	−1.012812873	0.347	0.837	1.2956E−31
KRT17P5	1.9894E−20	−1.01015447	0.368	0.714	4.008E−16
PRSS23	1.0446E−35	−1.004520785	0.564	0.961	2.1045E−31
B3GALNT1	5.0882E−28	−1.001859857	0.237	0.651	1.0251E−23
SYCE1	6.0571E−29	−0.993202541	0.344	0.765	1.2203E−24
PIP5K1B	1.6858E−37	−0.974347924	0.478	0.937	3.3964E−33
STOX1	1.6455E−22	−0.968211627	0.255	0.617	3.3152E−18
CD53	6.171E−31	−0.9625812	0.279	0.738	1.2433E−26
IDS	1.5236E−24	−0.958967284	0.422	0.783	3.0696E−20
ZNF560	2.7358E−26	−0.950568717	0.225	0.627	5.5118E−22
PPP1R14C	2.1546E−40	−0.939439473	0.649	0.991	4.3409E−36
FBLN2	2.1454E−20	−0.937438214	0.363	0.705	4.3222E−16
MSL1	2.1522E−21	−0.934180643	0.333	0.69	4.336E−17
ENTPD1	7.4728E−20	−0.915827694	0.384	0.711	1.5055E−15
PIGT	3.9939E−36	−0.901035468	0.639	0.97	8.0464E−32
SLC35B2	4.256E−36	−0.875229251	0.698	0.955	8.5745E−32
RNASE1	2.716E−22	−0.860773915	0.494	0.837	5.472E−18
LBH	1.0418E−21	−0.846731505	0.513	0.834	2.0989E−17
MROH8	3.2199E−23	−0.84450453	0.237	0.62	6.4871E−19
TEKT4P2	7.0669E−28	−0.844208534	0.232	0.648	1.4238E−23
LOC653786	2.0543E−17	−0.840282822	0.302	0.611	4.1387E−13
ZNF264	4.0733E−14	−0.825770152	0.347	0.614	8.2064E−10
H2AFY	2.0712E−20	−0.814941568	0.461	0.798	4.1729E−16

PEAK1	1.018E−19	−0.812122919	0.295	0.645	2.051E−15
DDX4	4.3716E−32	−0.809024217	0.684	0.97	8.8076E−28
C9orf156	2.7889E−17	−0.804487435	0.382	0.696	5.6188E−13
HACD4	1.7688E−19	−0.794728412	0.281	0.636	3.5635E−15
SNX27	6.5307E−29	−0.778815375	0.836	0.973	1.3157E−24
DCAF4L1	1.2182E−21	−0.776590454	0.689	0.952	2.4543E−17
MLLT3	4.0909E−21	−0.775763195	0.253	0.608	8.2419E−17
CTSH	6.8461E−22	−0.771850354	0.438	0.786	1.3793E−17
SGSM2	8.0206E−18	−0.766648405	0.375	0.723	1.6159E−13
ANKRD20A12P	1.6652E−14	−0.75809797	0.44	0.699	3.3548E−10
LINC00467	2.3077E−28	−0.75584772	0.651	0.955	4.6492E−24
DAZL	3.6891E−38	−0.755059147	0.81	0.997	7.4325E−34
MARCH8*	5.4784E−26	−0.749775179	0.649	0.925	1.1037E−21
SERPING1	1.4649E−28	−0.749279779	0.918	0.994	2.9514E−24
PER3	1.5315E−23	−0.747973104	0.283	0.666	3.0856E−19
TJP3	6.9908E−23	−0.744473926	0.637	0.892	1.4084E−18
STK39	1.1814E−18	−0.743047536	0.532	0.892	2.3801E−14
RAB6B	1.1106E−19	−0.74270123	0.333	0.69	2.2375E−15
ZNF473	2.4975E−21	−0.742492915	0.532	0.855	5.0318E−17
HDAC9	2.657E−24	−0.741574136	0.548	0.898	5.3531E−20
PTCH1	2.1952E−22	−0.740849297	0.267	0.657	4.4227E−18
ICA1	3.4635E−27	−0.739654736	0.33	0.783	6.978E−23
LMBR1L	1.8688E−23	−0.734934697	0.398	0.819	3.765E−19
LINC00339	5.3781E−19	−0.733037619	0.279	0.627	1.0835E−14
COL22A1	1.5608E−13	−0.732414689	0.525	0.765	3.1445E−09
AGGF1	5.6422E−30	−0.731540303	0.693	0.964	1.1367E−25
STK31	5.2834E−15	−0.728976875	0.436	0.723	1.0645E−10
TDRD9	1.0273E−23	−0.716849627	0.393	0.771	2.0697E−19
SKAP2	6.3066E−27	−0.715284023	0.621	0.922	1.2706E−22
NR1H2	6.8461E−24	−0.707402344	0.719	0.952	1.3793E−19
RAB11B	3.0624E−20	−0.705575752	0.604	0.877	6.1699E−16
CDH2	1.4899E−26	−0.70375383	0.775	0.979	3.0017E−22
RHOG	1.5037E−21	−0.700048529	0.4	0.786	3.0296E−17
C2orf74	8.9902E−25	−0.699532446	0.646	0.925	1.8113E−20
PRR15	7.7557E−14	−0.69907575	0.344	0.614	1.5625E−09
RPRM	2.4825E−22	−0.698793285	0.527	0.873	5.0016E−18
ZNF16	1.6854E−16	−0.696337515	0.335	0.645	3.3957E−12
ZNF879	7.6354E−17	−0.695976082	0.326	0.648	1.5383E−12
SETD9	5.9144E−18	−0.694565159	0.319	0.66	1.1916E−13
SYAP1	1.0356E−25	0.696128406	0.934	0.916	2.0864E−21
NKAP	1.1532E−19	0.698613682	0.888	0.822	2.3233E−15
CETN2	1.2207E−45	0.698858231	0.995	0.991	2.4593E−41
LGALS1	1.357E−21	0.708947692	0.991	0.985	2.734E−17
FHL2	5.686E−10	0.715162506	0.724	0.605	1.1455E−05
UPF3B	2.5064E−31	0.719652309	0.948	0.94	5.0497E−27
DPPA5	5.8083E−12	0.719977419	0.925	0.937	1.1702E−07

CLDN7	1.3322E−08	0.726268983	0.649	0.524	0.0002684
THOC2	6.8972E−44	0.740649866	1	0.958	1.3896E−39
TSR2	1.1666E−32	0.741558609	0.979	0.976	2.3503E−28
CYB5A	9.2881E−19	0.746803755	0.916	0.861	1.8713E−14
TMSB4X	1.4802E−55	0.747375538	1	1	2.9822E−51
PPP1R15A	5.7929E−22	0.750559833	0.911	0.825	1.1671E−17
PYGL	8.0558E−09	0.756942769	0.726	0.675	0.0001623
OGT	1.0798E−26	0.757069373	0.965	0.931	2.1754E−22
GSPT2	3.7759E−08	0.7613267	0.637	0.539	0.00076073
PLIN2	1.1036E−23	0.76935916	0.925	0.825	2.2235E−19
PCSK1N	2.5619E−18	0.772128697	0.984	0.994	5.1614E−14
ATRX	4.2127E−37	0.776073115	0.991	0.967	8.4874E−33
SLC25A5	1.7795E−57	0.777908651	1	1	3.5851E−53
RNF113A	3.3715E−14	0.790091395	0.735	0.578	6.7926E−10
QTRT1	3.0617E−15	0.791055784	0.803	0.741	6.1684E−11
ATP11C	5.8543E−45	0.79455939	0.986	0.985	1.1795E−40
NMT2	3.5803E−13	0.7985535	0.785	0.645	7.2132E−09
ATP5G1	6.4042E−45	0.805022199	0.993	0.982	1.2903E−40
PDHA1	5.6829E−20	0.818387702	0.862	0.69	1.1449E−15
TCEAL4	2.9675E−20	0.829049098	0.885	0.795	5.9787E−16
EIF1AX	1.7764E−83	0.83421497	0.998	1	3.5789E−79
DBI	4.1487E−24	0.84088565	0.937	0.904	8.3583E−20
DMD	6.5097E−55	0.844933547	0.988	0.973	1.3115E−50
PHPT1	4.1446E−51	0.848538631	0.995	0.988	8.3502E−47
ALPPL2	3.904E−11	0.8531822	0.71	0.566	7.8654E−07
FAM60A	4.9018E−40	0.857126872	0.967	0.955	9.8757E−36
PRPS1	8.0598E−17	0.860180539	0.827	0.726	1.6238E−12
NUSAP1	1.5623E−15	0.862297448	0.965	0.919	3.1476E−11
LPCAT4	1.0852E−07	0.863853915	0.618	0.5	0.00218628
SLC2A1	1.5067E−12	0.879870181	0.721	0.566	3.0356E−08
CRYM	5.7288E−10	0.880329163	0.625	0.458	1.1542E−05
IFITM1	1.2316E−35	0.884027673	0.993	0.994	2.4812E−31
DIAPH2	7.7274E−28	0.908010669	0.904	0.774	1.5568E−23
HMGB3	1.1329E−56	0.909929067	0.995	1	2.2824E−52
HSD17B10	1.3383E−74	0.915124575	0.993	1	2.6962E−70
PI4KAP2	2.3698E−12	0.915855296	0.71	0.566	4.7744E−08
REC8	9.3001E−24	0.916568833	0.855	0.672	1.8737E−19
NDUFA1	1.3151E−90	0.920445929	1	1	2.6495E−86
APOC1P1	2.9874E−13	0.932229235	0.611	0.364	6.0188E−09
IDH3G	5.0253E−24	0.935794377	0.845	0.714	1.0124E−19
ATP6AP2	7.8391E−62	0.938017704	0.998	0.991	1.5794E−57
PRKX	7.7501E−16	0.940454002	0.609	0.343	1.5614E−11
ACTG2	6.2774E−25	0.948798301	0.951	0.864	1.2647E−20
BNIP3	2.5315E−40	0.950246556	0.998	0.997	5.1001E−36
STAG2	3.4495E−30	0.952531627	0.92	0.816	6.9498E−26
FKBP11	3.8911E−16	0.953011951	0.749	0.645	7.8394E−12

MDK	3.6426E−57	0.953998575	1	0.997	7.3388E−53
PIN4	1.7194E−74	0.97341047	0.991	0.994	3.4642E−70
RHOBTB3	2.9358E−11	0.977806015	0.616	0.431	5.9148E−07
SAT1	1.1857E−61	1.000273302	1	1	2.3887E−57
COL1A2	1.3021E−15	1.011314823	0.639	0.467	2.6234E−11
COX7B	1.3563E−64	1.012192301	0.993	0.988	2.7325E−60
BRDT	2.0261E−36	1.02425211	0.92	0.744	4.0819E−32
PGK1	8.3864E−76	1.030532987	0.998	1	1.6896E−71
NLRP7	2.6231E−30	1.039416876	0.93	0.828	5.2848E−26
CRIP1	8.8147E−55	1.048008315	0.988	0.946	1.7759E−50
DEPTOR	1.996E−15	1.05139215	0.637	0.434	4.0214E−11
ELF3	1.3192E−19	1.075156533	0.674	0.395	2.6578E−15
HSPB1	1.7833E−08	1.085057722	0.979	0.973	0.00035929
EIF2S3	3.4857E−65	1.095885869	0.998	0.979	7.0226E−61
LAS1L	8.0952E−64	1.111708673	0.977	0.922	1.6309E−59
LDHA	4.0217E−58	1.219874914	1	0.997	8.1026E−54
MEG3	3.4804E−66	1.239552588	0.998	0.997	7.0121E−62
SLC35F2	1.1048E−23	1.249868091	0.689	0.395	2.2259E−19
PRDX2	1.143E−46	1.25055862	0.934	0.798	2.3028E−42
GDPD2	3.9745E−24	1.281205743	0.785	0.56	8.0074E−20
ID1	2.8218E−30	1.403248743	0.878	0.846	5.6851E−26
ARRDC3	1.1065E−21	1.463934874	0.719	0.527	2.2292E−17
NGFRAP1	1.1167E−27	1.481777737	0.834	0.714	2.2498E−23
TUBB2B	4.731E−31	1.65301173	0.707	0.377	9.5316E−27
ANXA2	1.1838E−63	1.768904759	0.934	0.617	2.385E−59
PTN	1.159E−36	1.8157341	0.66	0.253	2.335E−32
CA2	1.13E−33	1.845621888	0.696	0.349	2.2766E−29
LINC01356	9.7734E−37	2.587518332	0.696	0.331	1.969E−32
